# Life and Death Decisions in the CD95 System: Main Pro-and Anti-Apoptotic Modulators

**Published:** 2009-04

**Authors:** Inna N. Lavrik, Peter H. Krammer

**Affiliations:** 1 Division of Immunogenetics, Tumorimmunology Program German Cancer Research Center, Im Neuenheimer Feld 280, D-69120 Heidelberg, Germany

## Abstract

Apoptosis is common to all multicellular organisms. Apoptosis can be triggered by the extrinsic (death receptor (DR)) or the intrinsic (mitochondrial) death pathways. CD95 (APO-1/Fas) is a prototypic member of the DR family. This review is focused on the mechanisms of CD95 (APO-1/Fas)-mediated apoptosis and the role in the apoptosis of the death effector domain (DED)-containing proteins: pro-apoptotic protein procaspase-8 and anti-apoptotic protein c-FLIP. Gaining insights into these processes will improve our understanding of the pathogenesis of diseases such as cancer, autoimmunity and AIDS, and will open new approaches to rational treatment strategies.

## Introduction: CD95 and CD95 signaling

CD95 (also called APO-1; Fas; fas antigen; tumor necrosis factor receptor superfamily member 6, TNFRSF6 or apoptosis antigen 1, APT1) is a member of the death receptor (DR) family, a subfamily of the tumor necrosis factor receptor superfamily (1). All members of the DR family are characterized by a cytoplasmic region termed the Death Domain (DD) (2; 3). DD are 80-100 amino acid long motifs involved in the transduction of the apoptotic signal. The DD belongs to the so-called 'death domain-fold superfamily'. This superfamily comprises the death domain (DD), the death effector domain (DED), and the caspase recruitment domain (CARD). Each of these motifs interacts with other proteins through homotypic interactions. All members of the DD-fold superfamily are characterized by similar structures that comprise six or seven antiparallel amphipatic α-helices. 

**Fig. 1.**
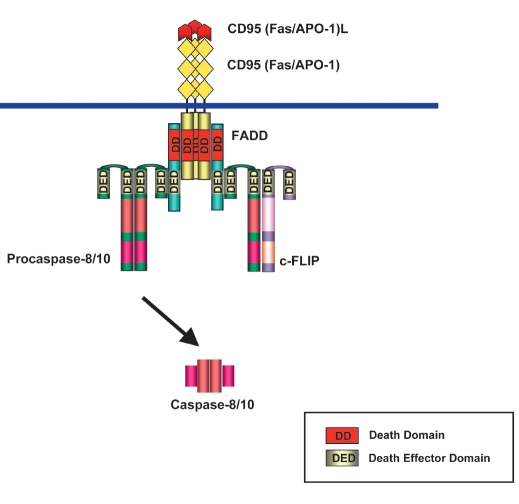
The CD95 death-inducing signaling complex (DISC). The DISC consists of CD95, (depicted in yellow), FAS-associated death domain, FADD, (depicted in light blue), procaspase-8/procaspase-10, (depicted in green), and cellular FLICE-inhibitory proteins, c-FLIP, (depicted in violet). DD are shown in red; DED are shown in light yellow. The interactions between the molecules at the DISC are based on homotypic contacts. The death domain (DD) of CD95 interacts with the DD of FADD while the death effector domain (DED) of FADD interacts with the N-terminal tandem DEDs of procaspase-8, procaspase-10 and c-FLIP.

Crosslinking of CD95 with its natural ligand, CD95L (CD178) (4), or with agonistic antibodies, such as anti-APO-1 (5), induces apoptosis in sensitive cells. The binding of CD95L or agonistic antibodies to CD95 leads to the formation of the receptor complex at the cellular membrane, which was named death-inducing signaling complex (DISC) (6). The DISC consists of oligomerized receptors, the DD-containing adaptor molecule FADD/MORT1 (Fas-Associated Death Domain), procaspase-8 (FLICE, MACHα, Mch5), procaspase-10, and the cellular FLICE-inhibitory proteins (c-FLIP) (Fig. 1) (7-9). The interactions between the molecules at the DISC are based on homotypic contacts. The DD of the receptor interacts with the DD of FADD, while the DED of FADD interacts with the N-terminal tandem DEDs of procaspases-8, -10 and c-FLIP. As a result of DISC formation procaspase-8 is activated at the DISC resulting in the formation of the active caspase-8. Caspase-8 cleaves and thereby activates downstream effector caspases-3, -6, and -7. This is followed by the cleavage of caspase substrates, which comprise a number of cellular proteins playing a central role for the normal functioning of the cell leading to demolition of the cell. 

DED proteins at the DISC play a central role in the regulation of DR-induced apoptosis. Recruitment of procaspase-8 to the DISC, followed by its activation at the DISC and formation of active caspase-8 heterotetramers, triggers the apoptotic pathway. Recruitment of c-FLIP proteins to the DISC has the opposite effect: c-FLIP proteins block procaspase-8 activation at the DISC and thereby apoptosis induction. Therefore, life/death decisions at the DISC are defined by the balance of two DED proteins: procaspase-8 and c-FLIP. In this review we describe in detail the DED-proteins procaspase-8 and c-FLIP and the mechanism of their pro- and anti-apoptotic action.

## Proapoptotic DED proteins of the DISC: Procaspase-8 as a member of the caspase family

**Fig. 2.**
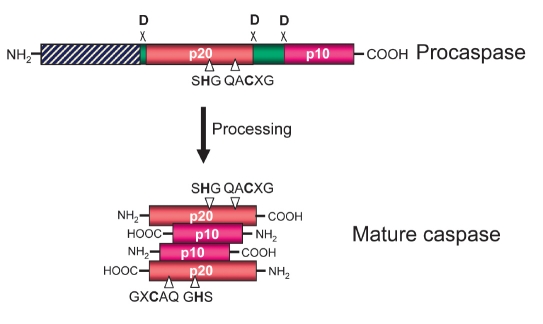
Scheme of procaspase activation. Cleavage of the procaspase at the specific Asp-X bonds leads to the formation of the mature caspase that comprises the heterotetramer p202-p102 and the release of the prodomain. The residues involved in the formation of the active center are shown.

**Fig. 3.**
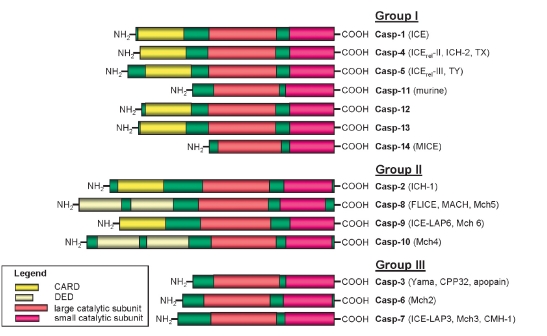
The caspase family. Three major groups of caspases are presented. Group I: inflammatory caspases. Group II: apoptosis initiator caspases. Group III: apoptosis effector caspases. The caspase activation recruitment domain (CARD), the death effector domain (DED), the large (p20), and the small (p10) catalytic subunits are indicated.

Procaspase-8 (FLICE, MACHα, Mch5) belongs to the family of caspases (7; 10). Caspases, a family of cysteinyl aspartate specific proteases, are synthesized as zymogens with a prodomain of variable length followed by a large subunit (p20) and a small subunit (p10). Caspases are activated through proteolysis at specific aspartate (D) residues that are located between the prodomain, the p20, and p10 subunits (Fig. 2) (11). This results in the generation of mature active caspases that consist of heterotetramers p202-p102. Subsequently, active caspases specifically process various substrates that are involved in apoptosis and inflammation. Depending on their function and the structure of the prodomain, caspases are divided into initiator caspases and effector caspases and are typically divided into three major groups (Fig. 3) (11). The caspases with large prodomains are referred to as inflammatory caspases (group I) and initiator of apoptosis caspases (group II), while caspases with a short prodomain of 20-30 amino acids are named effector caspases (group III). 

All caspases are produced in cells as catalytically inactive zymogens and must undergo proteolytic processing and activation during apoptosis (12). The effector caspases are activated by initiator caspases. In turn, initiator caspase activation takes place in large protein complexes bringing together several caspase zymogens. All initiator caspases are characterized by the presence of a member of the 'death domain-fold superfamily' (DED or CARD), which enables their recruitment into their initiation complexes. Procaspases-8 and -10 possess two tandem DEDs in their prodomain (Fig. 3). The CARD is found in procaspases-1, -2, -4, -5, -9, -11, and –12 (Fig. 3). DEDs and CARDs are responsible for the recruitment of initiator caspases into death- or inflammation-inducing signaling complexes resulting in proteolytic autoactivation of caspases that subsequently initiates inflammation or apoptosis. 

**Fig. 4.**
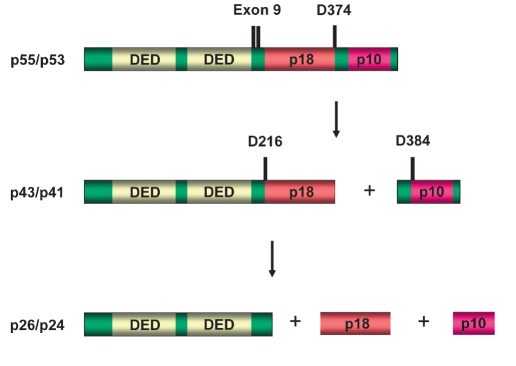
Scheme of procaspase-8 and the two-step mechanism of procaspase-8 activation. Procaspase-8a/b (p55/p53) is shown in green; DEDs are presented in light yellow. The N-terminal cleavage products: p43/p41 and prodomains p26/p24 as well as caspase-active domains: p18 and p10 are indicated. Two cleavage steps with the resulting products are presented.

Procaspase-8 is activated at the DISC (13). Two isoforms of procaspase-8 (procaspase-8a and procaspase-8b) were reported to be bound to the DISC (14). Both isoforms possess two tandem DED domains, as well as the catalytic subunits p18 and p10 (Fig. 4). Procaspase-8a contains an additional 2 kDa (15 aa) fragment, which results from the translation of exon 9. This small fragment is located between the second DED and the large catalytic subunit resulting in the different length of procaspase-8a (p55) and, consequently, procaspase-8b (p53).

The activation of procaspase-8 is believed to follow an 'induced proximity' model in which high local concentrations and favourable mutual orientation of procaspase-8 molecules at the DISC lead to their autoproteolytic activation (15). There is strong evidence from several in vitro studies that autoproteolytic activation of procaspase-8 occurs after oligomerization at the receptor complex (16). Furthermore, dimerization of two procaspase-8 molecules at the DISC has been shown to be necessary for procaspase-8 activation (17). Procaspase-8a/b at the DISC undergoes autocatalytic cleavage, for which a two-step mechanism has been described (Fig. 4) (14; 18). The initial cleavage at Asp374 generates the two subunits p43/p41 and p12. In a second step, cleavage takes place at Asp216 and Asp384, producing the active enzyme subunits p18, p10 and the prodomains p26/p24. As a result of procaspase-8 processing, the caspase-8 heterotetramer (p18/p10)2 starts the apoptotic signaling cascade (19).

## Anti-apoptotic DED-protein of the DISC: Cellular FLICE-inhibitory proteins (c-FLIP)

**Fig. 5.**
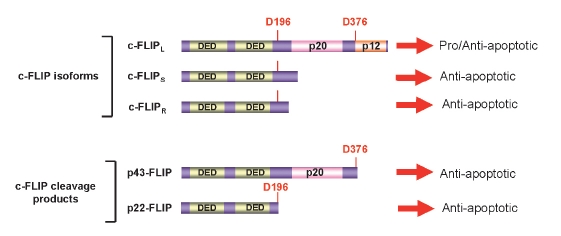
The scheme of c-FLIP proteins. c-FLIP isoforms and c-FLIP cleavage products are shown. DED (Death effector domains) and caspase-like domains (p20 an p12) are indicated. D376 and D196, leading to the generation of p43-FLIP and p22-FLIP, respectively, are presented in red.

c-FLIP, also known as FLAME-1/I-FLICE/CASPER/CASH/MRIT/CLARP/Usurpin, is a well-described inhibitor of DR-mediated apoptosis. The current view on c-FLIP proteins is shown in figure 5. Five c-FLIP proteins have been characterized so far: three c-FLIP isoforms and two cleavage products (9; 20-24). The three c-FLIP isoforms comprise: Long (L), Short (S), and Raji (R), e.g. c-FLIPL, c-FLIPS, and c-FLIPR, respectively (Fig. 5). All three isoforms possess two DED domains and thereby bind to the DISC. In this way, the short FLIP isoforms, c-FLIPS, and c-FLIPR block procaspase-8 activation and apoptosis. The role of the long c-FLIP isoform, c-FLIPL, at the DISC is controversial. It has been shown that depending upon its concentration at the DISC it can act either as an anti-apoptotic molecule, functioning in a way analogous to c-FLIPS, or as a pro-apoptotic molecule, facilitating the activation of procaspase-8 at the DISC (25; 26). This pro-apoptotic role is in agreement with the phenotype of c-FLIP-deficient mice, which are characterized by heart failure and death at embryonic day 11 (27).

In addition, two cleavage products of c-FLIP have been reported until now: p43-FLIP and p22-FLIP (9; 24). P43-FLIP was shown to be generated from c-FLIPL at the CD95 DISC as a result of procaspase-8 cleavage at D376. P22-FLIP was shown to be the N-terminal cleavage product of c-FLIP resulting from procaspase-8 cleavage at D196. In contrast to p43-FLIP, p22-FLIP is formed in the cytosol independently of DR-stimulation. In addition, p22-FLIP turned out to be a prominent inducer of NF-κB activity by binding to the IKK complex. 

## Regulation of life and death by DED proteins

**Fig. 6.**
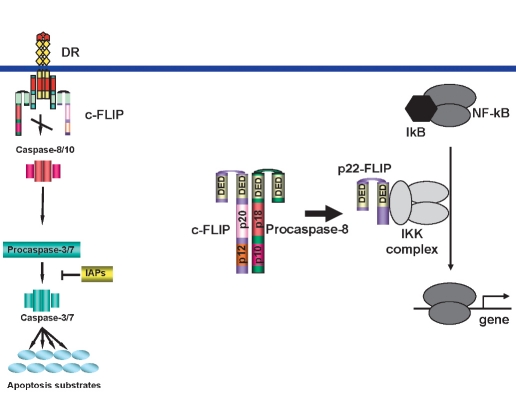
DED proteins: procaspase-8 and c-FLIP at the DISC and in the cytosol. c-FLIP proteins block procaspase-8 activation at the DISC (left side). Procaspase-8 and c-FLIP form dimers in the cytosol leading to generation of p22-FLIP. P22-FLIP binds to IKK complex via IKKγ, which leads to the induction of NF-κB (right side).

DED proteins procaspase-8 and c-FLIP play a central role in the regulation of DR-induced apoptosis and might also induce the NF-κB pathway (6). Regulation of DR-induced apoptosis by procaspase-8 and c-FLIP occurs at the DISC. (Fig. 6. left side). Procaspase-8 is activated at the DISC inducing the apoptotic process, while this activation can be inhibited by all reported c-FLIP proteins. The only exception is the c-FLIPL isoform, which might induce procaspase-8 activation when expressed at low concentrations and block procaspase-8 activation when expressed at high concentrations. Therefore, procaspase-8 at the DISC has a pro-apoptotic role and c-FLIP proteins, except for the c-FLIPL isoform, possess an anti-apoptotic function.

Interestingly, in the cytosol, interactions between procaspase-8 and c-FLIP have been reported to induce the NF-κB pathway rather than apoptotic pathways. Recently, a new NF-κB-activating pathway initiated by procaspase-8 has been described. It has been shown that, independently of DR stimulation, non-apoptotic procaspase-8 activity generates the p22-FLIP cleavage product that leads to the induction of NF-κB (Fig. 6, right side). The role of procaspase-8 in this pathway is different from its pro-apoptotic activity at the DISC. Procaspase-8 does not undergo processing leading to apoptosis induction with active heterotetramer formation but rather utilizes its so-called proform activity processing c-FLIP to the p22-FLIP cleavage product. It is likely that procaspase-8 constitutively forms heterodimers with c-FLIP cleaving c-FLIP to p22-FLIP. Thus, the ratio between procaspase-8 and c-FLIP in cells would be the crucial factor defining the amount of generated p22-FLIP and, correspondingly, the potential to induce NF-κB. 

The balance between DED-containing proteins may provide sensitive signaling check points that cells use for signaling cross-talk and switching between apoptosis-resistant and sensitive phenotypes and, thus, between life and death. Furthermore, the balance between DED-proteins also depends on the subcellular localization. The regulation by c-FLIP and procaspase-8 of life/death decisions at the DISC is different from the cytosolic events. At the DISC, c-FLIP mostly acts as a devoted procaspase-8 inhibitor, while in the cytosol it uses procaspase-8 activity to initiate cleavage to p22-FLIP and the subsequent NF-κB induction (Fig. 6). Consequently, procaspase-8 initiates apoptosis at the DISC and NF-κB in the cytosol. The crosstalk between the DED proteins in the cytosol and at the DISC will be a topic of future studies.
